# Quantifying suitable late summer brood habitats for willow ptarmigan in Norway

**DOI:** 10.1186/s12898-018-0196-6

**Published:** 2018-10-03

**Authors:** Mikkel Andreas Jørnsøn Kvasnes, Hans Christian Pedersen, Erlend Birkeland Nilsen

**Affiliations:** Norwegian Institute for Nature Research, Torgarden, P.O.Box 5685, Trondheim, 7485 Norway

**Keywords:** Resource selection function, Distance sampling, Line transect survey, Habitat suitability map, Predictions

## Abstract

**Background:**

Habitat models provide information about which habitat management should target to avoid species extinctions or range contractions. The willow ptarmigan inhabits alpine- and arctic tundra habitats in the northern hemisphere and is listed as near threatened (NT) in the Norwegian red list due to declining population size. Habitat alteration is one of several factors affecting willow ptarmigan populations, but there is a lack of studies quantifying and describing habitat selection in willow ptarmigan. We used data from an extensive line transect survey program from 2014 to 2017 to develop resource selection functions (RSF) for willow ptarmigan in Norway. The selection coefficients for the RSF were estimated using a mixed-effects logistic regression model fitted with random intercepts for each area. We predicted relative probability of selection across Norway and quantile-binned the predictions in 10 RSF bins ranging from low-(1) to high-(10) relative probability of selection.

**Results:**

Random cross-validation suggest that our models were highly predictive, but validation based spatial blocking revealed that the predictability was better in southern parts of Norway compared to the northernmost region. Willow ptarmigan selected for herb-rich meadows and avoided lichen rich heathlands. There was generally stronger selection for vegetation types with dense field layer and for rich bogs and avoidance of vegetation types with sparse field layer cover and for lowland forest. Further, willow ptarmigan selected for areas around the timberline and for intermediate slopes. Mapping of the RSF showed that 60% of Norway is in the lowest ranked RSF bin and only 2% in the highest ranked RSF bin.

**Conclusions:**

Willow ptarmigan selected for vegetation types with dense field layer and bogs at intermediate slopes around the timberline. Selection coincides with previous habitat selection studies on willow ptarmigan. This is the first attempt to assess and quantify habitat selection for willow ptarmigan at a large scale using data from line transect distance sampling surveys. Spatial variation in predictability suggests that habitat selection in late summer might vary from north to south. The resource selection map can be a useful tool when planning harvest quotas and habitat interventions in alpine areas.

## Background

Knowledge about patterns of habitat selection is often needed in order to make evidence-based management decisions. For instance, Smereka et al. [1] mapped the relative probability of selection for den sites for grizzly bears (*Ursus arctos horibilis*) in the Mackenzie Delta, Northwest Territories, Canada, to reduce human-bear conflicts by guiding human activity and land-use. A habitat selection model [2] has also been applied to harvest management of willow ptarmigan (*Lagopus lagopus*) in Northern Norway, where quotas are estimated based on a combination of pre-harvest densities and the amount of suitable habitat for willow ptarmigan available within the hunting area. Although a plethora of methods have been developed to assess patterns of habitat selection, resource selection functions (RSF) [3] are among the most frequently used methods to model habitat selection in animals (e.g., [1, 4–6]). An RSF is a function that is proportional to the probability of selection by an animal (c.f. [7, 8]) and is estimated directly from data. Data in the context of RSF’s is usually a set of locations where individuals are observed and a set of randomly generated available locations where the individuals could have been observed (used vs. available units). Variables associated with the observations may be habitat variables or covariates like elevation, topology, vegetation types or human-disturbance and infrastructure metrics. In a presence/available framework, variables that are assumed important for habitat selection are compared at the locations of the observations and the locations of available sites. A predictive RSF can be mapped and used to predict the relative selection for different geographical units based on their environmental characteristics [3, 7]. RSFs have been developed for many wildlife species [1, 2, 4, 5, 9, 10], often with the purpose to quantify suitable habitat for species that are of conservation concern or to answer questions related to the ecological dynamics of the system.

Willow ptarmigan is a medium-sized grouse species distributed in tundra habitats and boreal forests in the northern hemisphere [11]. Willow ptarmigan inhabits treeless alpine- and arctic tundra habitats most of the snow-free season, but it also occurs in northern boreal forests near tundra habitats. In Norway, it was recently listed as near threatened (NT) in the national red list of species [12], due to a 15–30% decline in the breeding population during the last decade (e.g., [13]). Predation, climate change, unsustainable harvest and potentially loss of habitat are all assumed to have contributed to the observed decline, but the relative contribution from these effects is to date not quantified [12].

There has been a rapid development of human infrastructure in the tundra areas of Norway during the nineteenth century, mainly due to construction of hydroelectric power installations, recreational facilities and roads. Moa et al. [14] found that autumn densities of willow ptarmigan were generally higher in management units with high proportion of areas located far from human infrastructure (see also [15, 16]). This suggests that habitat alteration affects willow ptarmigan populations. There is however a lack of studies quantifying and describing within range habitat selection for willow ptarmigan (but see [10, 17–20]). Some studies describing habitat selection however, have shown that willow ptarmigan broods, in general select for rich bogs close to willow-(*Salix* spp.) and dwarf birch (*B. nana*) thickets and avoid dry and sparsely vegetated habitats like heaths during summer and early autumn [10, 19, 20]. Willow ptarmigan is an indicator species for biodiversity in Norwegian mountains [21] and its distribution overlap with several other alpine and boreal species [13, 22]. Hence, it is possible that willow ptarmigan can play a role as an umbrella species in the alpine ecosystems, especially because of its status as an economically important game species [23].

In this study, we model willow ptarmigan habitat selection in August using exponential RSF’s. The exponential RSF was estimated using a mixed effects logistic regression model with a presence/available design. This study is the first objective attempt to model habitat selection for willow ptarmigan across Norway. We use satellite-based vegetation maps, slope and aspect from a digital elevation model and timberline measures as predictor variables. Based on previous studies of willow ptarmigan, we predict that willow ptarmigan selects vegetation types that offers food and concealment against predators. In addition, we predict that willow ptarmigan selects for areas close to the timberline, due to the special adaptation to alpine and sub-alpine areas during the snow-free season. We further expect that willow ptarmigan avoid steep slopes. Southernly exposed slopes have normally more vigorous plant communities than northerly slopes due to prolonged sun-exposure, so we also expect that willow ptarmigan select for southern slopes rather than northern slopes.

## Methods

### Study area

**Fig. 1:**
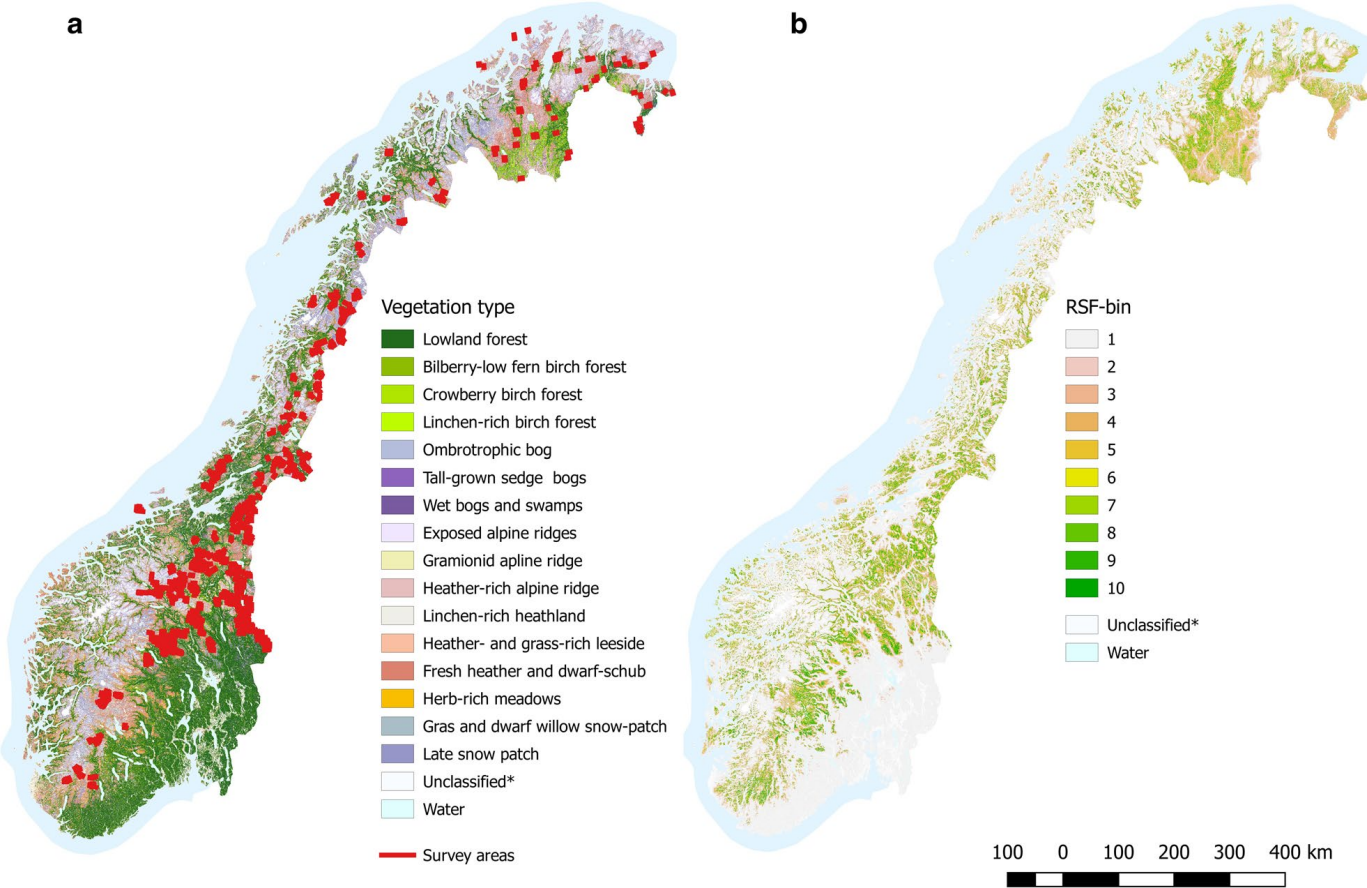
Study area showing **a** the distribution of survey areas and vegetation types, **b** the relative probability of selection for willow ptarmigan during late summer/early autumn, ranging from low relative probability of selection (1) to high relative probability of selection (10)

Data from survey areas distributed in alpine tundra, low arctic tundra and northern boreal forests throughout Norway (Fig. 1a) was used as a basis for our RSF. The vegetation in the tundra is dominated by small and medium-sized shrubs [e.g., willows, dwarf birch, and heath (*Vaccinium* spp. and *Caluna* spp.)]. The northern boreal forests are dominated by mountain birch (*B. pubescens*), Scots pine (*Pinus sylvestris*), and Norway spruce (*Picea abies*). The vertebrate fauna is dominated by large ungulates like wild- and semi-domestic reindeer (*Rangifer tarandus*) and moose (*Alces alces*), rodents (e.g., *Microtus* spp.), and terrestrial birds. Important predators on willow ptarmigan include red fox (*Vulpes vulpes*), stoat (*Mustela erminea*), pine marten (*Martes martes*), gyrfalcon (*Falco rusticolus*), rough-legged buzzard (*Buteo lagopus*) and golden eagle (*Aquila chrysaetos*). Livestock grazing by sheep and cattle is common in many areas during summer (Jun–Aug). Human population density is generally low within these areas. However, some areas are located in proximity to larger villages and in some areas, there are several cabin villages and isolated cabins, many of which are only seasonally inhabited.

### Data collection

**Fig. 2:**
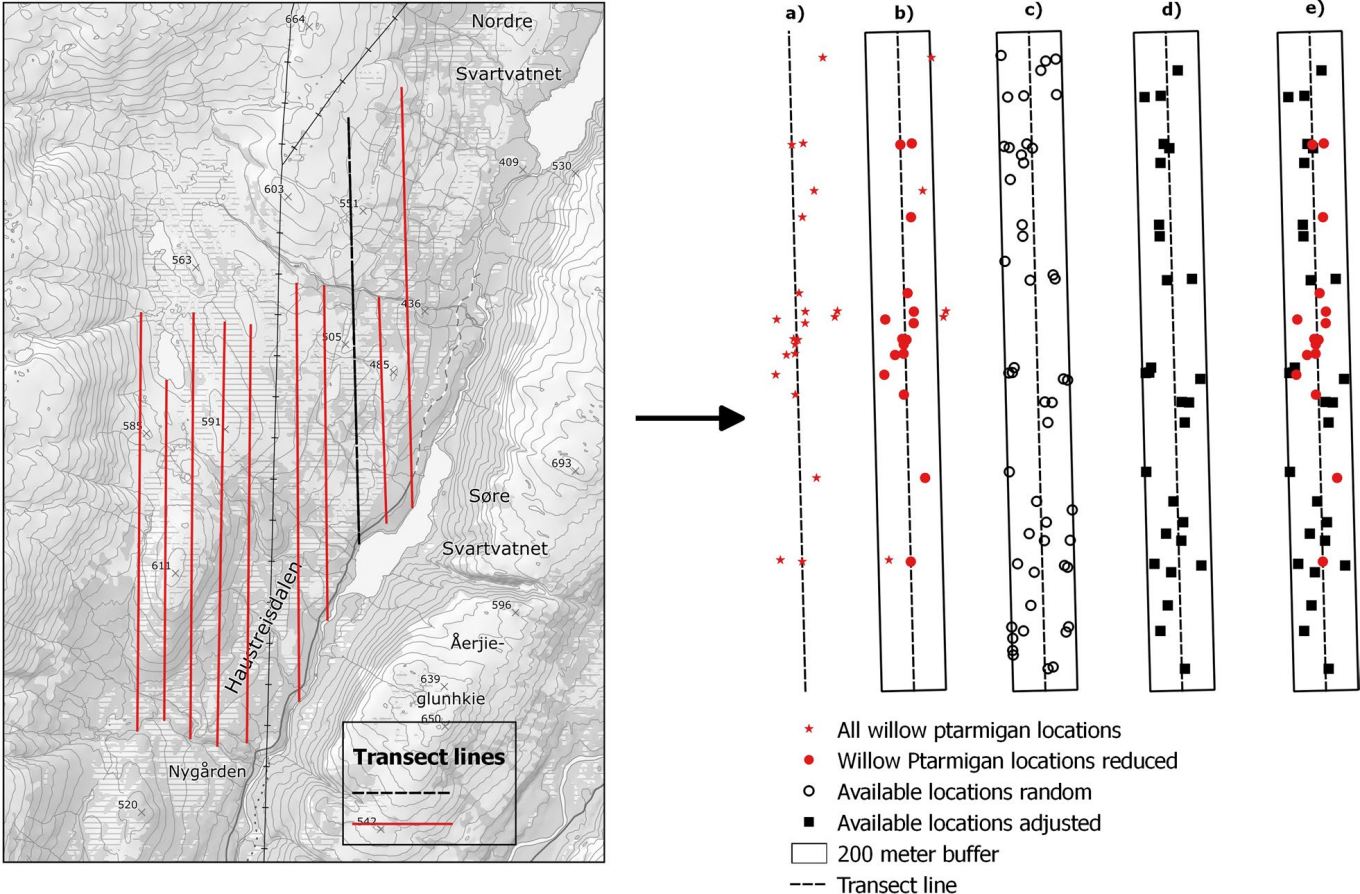
Example area with transect-lines observations and random locations. To the left: Transect-lines placed in the landscape. On the right; **a** is a transect-line with all observations from 2014 to 2017, **b** the transect-line and observations with a 200-m buffer. Observations outside the 200-m buffer and observations with > 30-m deviation between reported and estimated perpendicular distance were excluded from analyses (see “Methods”). **c** Randomly generated available locations within the 200-m buffer, **d** available locations after adjusting for detection probability (see “Methods”) and **e** is willow ptarmigan locations and adjusted available locations used in the modelling

We used information about willow ptarmigan occurrence based on observations done along line-transect surveys from 2014 to 2017. Transects were in most areas spaced out systematically following map grids with a 500 m interval, and often either in north–south or east–west bearing (see Fig. 2a). The same transects were repeatedly surveyed each year. Following a distance sampling protocol [24] a dog handler with pointing dogs and an observer walked along the transect-lines with one free-ranging dog at a time searching both sides of the line [25, 26]. At each bird encounter, the observer recorded the species, total number of birds (juveniles, adult males, adult females, or birds of unknown sex or age), the perpendicular distance from the transect line to the observation, the geographical location (centre of the brood/covey if several birds) of the birds (in UTM) as well as the time of day the observation was made. The main purpose of the line-transect survey program is to estimate pre-harvest densities of willow ptarmigan in the survey areas for harvest management purposes. So, the survey areas are not chosen at random, but mainly driven by local initiatives and does not follow a strict design. However, the location-data collected during the surveys are also suitable for assessing habitat selection using resource selection functions [10]. Detailed description for the sampling protocol used for estimating willow ptarmigan densities is outlined in Pedersen et al. [27], Pedersen et al. [25] and Eriksen et al. [28].

All observation data from the line transects surveyed were registered by the field worker to “Hønsefuglportalen” (http://honsefugl.nina.no/Innsyn/ [29]), a common e-infrastructure and data portal supporting the line transect survey program in Norway. We used a JDBC-connection with Rstudio [30] and the library «RJDBC» [31] to download survey data from the SQLServer database. In total, 17,386 willow ptarmigan locations from 7923 surveys along 2543 distinct transect lines across 179 survey areas were downloaded. We established buffers covering 200 m on each side of the transect lines and we discarded all observations made outside the line-buffers (2778 used locations). The size of the buffers was set at 200 m because most of the used locations are within this distance from the transect-line (> 80%) and the procedure exclude observations that are either regarded as outliers or that have typo in the UTM coordinate text. Furthermore, based on the geographical position of the used locations and the geographical location of the transect line, we estimated perpendicular distances from transect lines to used locations and compared this to the perpendicular distance reported by the field workers. Whenever the deviation between these measures were > 30 m, we discarded the observation to optimize the quality of our data (cf. 30 * 30 m is the spatial resolution of the vegetation variables, see “*Vegetation variables*” below). Additional 2427 observations were omitted in this procedure (see Fig. 2 and Table 1 for details regarding data management).

**Table 1:** Summary of the process from importing data to the final dataset (see also “Methods” section)

Action	Used locations	Surveys^a^	Available locations	Transects	Survey areas
Imported data	17,386	7923	–	2543	179
Removed use locations > 200 m	14,608	7397	–	2496	179
Removed use locations > 30-m deviation	12,181	6927	–	2443	179
Removed surveys without use locations^b^	12,181	4875	–	2109	176
Generate random locations^c^	–	6927	66,174	2443	179
Adjusted random locations	–	–	38,485	2438	179
Removed non-habitat (final dataset)	12,146	–	38,149	2440	179

^a^Survey is a unique ID for each time a transect is surveyed

^b^The complete dataset of used locations excluded surveys without observations

^c^Generated available locations for each survey

#### Defining available habitat

To quantify the distribution of available habitat we generated random locations within the surveyed area. Based on a presence-available design (as in our case), observations are expected to be drawn from a sample of available locations [7]. Therefore, the randomly generated available locations should represent the available habitat within the area that is covered by the surveys. Since our observations are sampled along line transects it is expected by design that the detection probability decreases as a function of distance from the transect-line [10, 24, 27]. To achieve a proper distribution of available locations [7] we performed a four-step procedure following Kastdalen et al. [10] to achieve a similar probability distribution among observations and available locations. (1) Within the line-buffers, we generated random available locations (A_i_). The number of locations per line-buffer was set according to the effort (km transect) multiplied by 3 (e.g. 4 km transect surveyed in 4 years: 1 available location * 16 km * 3 = 48 random locations within the line-buffer). Following this procedure, we also generated available locations for transect lines without used locations. This resulted in 66,174 random available locations (A_i_). (2) We used the R library “distance” [32] to estimate half-normal detection functions (gx_o_) from the used locations (see above). (3) Then, gx_o_ was used to estimate the detection probability (O_i_) of each location A_i_ based on its distance from the transect line. O_i_ has a value between zero and one. (4) Finally, we generated a random number between zero and one (P_i_) for each A_i_. A_i_ was included in the set of available locations in the final analysis whenever P_i_ < O_i_. After omitting available locations in step four, our sample of available locations were 38,149 distributed with the same probability distribution as the real observations [10] (Fig. 2b).

### Predictor variables

#### Vegetation data

Vegetation type at willow ptarmigan locations and available locations was extracted from a digital raster-map with a resolution of 30 × 30 m (SatVeg [33]). This map consists of 25 generalized vegetation types [34] covering Norway (Fig. 1a and Table 2). Ten vegetation classes were classified as alpine, three were classified as bogs and open swamp vegetation, and eight as forest vegetation. The remaining four classes; water, agricultural areas, cities and built-up areas and unclassified or shadow affected areas were all considered as non-habitat. We kept alpine classes, bogs and swamps as original classes. We considered forest classes; Bilberry-(*Vaccinium myrtillus)*, Crowberry-(*Empetrum nigrum*) and Lichen-rich birch forests as mainly sub-alpine mountain birch forest and kept as original classes’ while we pooled the remaining forest classes into one class representing lowland forests dominated by coniferous tree species.

**Table 2:** Categorical and continuous landscape variables used to determine relative probability of selection for willow ptarmigan

Variable	Category	Units	Used sites	Available sites
Vegetation type	Exposed alpine ridges (# 12)	–	60	460
	Bilberry-low fern birch forest (# 6)		366	1481
	Wet mires, sedge swamps and reed beds (# 11)		147	633
	Fresh heather and dwarf-schub communities (# 17)		4410	11,380
	Graminoid apline ridge (# 13)		216	894
	Herb-rich meadows (# 18)		572	994
	Tall-grown lawn vegetation (# 10)		666	1767
	Crowberry birch forest (# 7)		267	959
	Lichen-rich birch forest (# 8)		222	929
	Lichen-rich heathland (# 15)		145	1063
	Heather- and grass-rich early snow patch communities (# 16)		503	1924
	Heather-rich alpine ridge (# 14)		2240	6955
	Lowland forest (# 1–5)		711	3620
	Bryophyte late snow patch vegetation (# 20)		207	1100
	Gras and dwarf willow snow-patch vegetation (# 19)		94	742
	Ombrotrophic bog and low-grown lawn vegetation (# 9)		1320	3248
Aspect	East	–	2920	8704
	Flat		72	360
	North		3246	10,227
	South		2991	9589
	West		2917	9269
Timberline	Over	–	6251	18,263
	Under		5895	19,886
Elevation	–	Meters	899 (6 to 1419)	861 (4 to 1501)
Aspect		Degrees	182 (0 to 360)	183 (0 to 360)
Slope		Degrees	5.23 (0 to 46.04)	5.02 (0 to 61.41)
Timberline (deviation)		Meters	2.70 (− 330 to 314)	− 5.20 (− 383 to 495)

Categorical variables are presented with the number of used sites and available sites, respectively. Continuous variables are presented with median and range of the variable for used sites and available sites, respectively. Numbers in parenthesis refers to the original vegetation type number in the vegetation map

#### Landscape data

We extracted aspect in degrees and slope in degrees from a digital elevation model (DEM) with a resolution of 10 × 10 m (Norwegian Mapping Authority: https://kartkatalog.geonorge.no/). We did not model the DEM (m.a.sl.) directly because of the clear bioclimatic gradients present in our large study area.

Aspect is a circular variable (0°—north to 360°—north) and was therefore transformed to radians $\left(r_{aspect} = aspect*\left( {\frac{2\pi }{360}} \right) \right)$, and in the next step we created two variables representing north-exposure $\left(N_{aspect} = { \cos }\left( {r_{aspect} } \right) \right)$ and eastern exposure ($\left(E_{aspect} = { \sin }\left( {r_{aspect} } \right) \right)$ [6]. We also constructed a categorical variable with five levels representing aspect, north (315°–45°), east (45°–135°), south (135°–225°), west (225°–315°) and flat areas (0°).

#### Timber line

To describe variation in altitude we used a raster-map with regional empirical timber line (RET) in meter above sea level, with a 100 × 100 m resolution [35]. To consider the bioclimatic variation caused by latitudinal and longitudinal gradients, we combined the DEM (see above) with the RET. Taking the regional empirical timber line at each location as the reference point (i.e. 0 m above the timberline) we calculated the deviation in altitude (meters) from the timberline to each willow ptarmigan location and available location.

All predictor variables at observations and available locations were extracted from the raster-maps using GRASS [36] and the function «r.what» through RStudio [30] with library «rgrass7» [37]. Summaries of the predictor variables are given in Table 2.

### Statistical analysis

#### Model development

We omitted used and available locations placed in “non-habitat” (35 used locations and 336 available locations, se definition of non-habitat above). Our final data set used for analyses of willow ptarmigan habitat selection consisted of 12,146 willow ptarmigan observations and 38,149 available locations (Table 1). We estimated selection coefficients for the RSF by comparing environmental conditions of used locations to available locations using a mixed-effects logistic regression model [38–40]. Observations and available locations are stratified by survey areas. However, there is unbalance in the data since the number of locations per survey area vary. Gillies et al. [39] showed that using a random intercept when data are unbalanced improve model fit greatly and can change the direction of model coefficients. Therefore, we used Generalized Linear Mixed Model (GLMM) and fitted random intercepts for each survey area. The mixed effect model with random intercepts allow us further to account for spatial variation in density (density, because available points are generated based on effort not number of observed locations in the survey areas) and it provide marginal selection coefficients that can be used to make predictions also outside the sampled area. This approach assume that the explanatory variables has the same effects across all survey areas, although density may vary between areas. We did not consider effects of sex- and brood size on patterns of habitat selection.

The coefficients ($\beta_{n} )$ estimated from the mixed-effect logistic regression model is the logarithmic relative selection strength (log-RSS) [41] for a given variable. We estimated the relative probability of selection at a given location using an exponential resource selection function (RSF):

1 $$w\left( x \right) = { \exp }\;\left( {\beta_{1} x_{1} + \beta_{2} x_{2} + \beta_{3} x_{3} + \beta_{4} x_{4} \ldots \beta_{n} x_{n} } \right),$$

where $w\left( x \right)$ is relative probability of selection at location *x* and $\beta_{1}$ thorough $\beta_{n}$ is the estimated relative selection strength for explanatory variables $x_{1}$ thorough $x_{n}$ from the logistic regression model. Note that we do not include random intercepts in the RSF since or objective is to predict relative probability of selection across the whole of Norway.

To facilitate model convergence, we standardized continuous variables (aspect, slope, deviation from the timberline) to zero mean and one standard deviation [42].

We evaluated different combinations of explanatory variables, but always including the variable vegetation type when developing the RSF. We also tested for quadratic effects of slope and deviation from the timberline, and we evaluated models including aspect either as one categorical variable with five levels (north, east, south, west and flat) or as two continuous variables (north–south and east–west).

#### Model selection and validation

Model selection was based on AIC (Akaike Information Criterion). We considered 24 model combinations and the model with lowest AIC is considered the best supported model, but ΔAIC < 2 suggest that models are statistically equivalent and thus equally supported by the data [43]. In such situations, we followed the principle of parsimony and selected the least complex model. The most parsimonious model was evaluated using k-fold cross-validation [7, 44]. K-fold cross-validation yield metrices to assess a model’s ability to predict high relative probability of selection at locations where the species are observed. We divided the dataset into 5 approximately equal-sized datasets (folds) using random k-fold portioning in the R-library “dismo” [45]. For each model under validation, the k-fold cross-validation procedure followed four steps (repeated for each of the fivefold). (1) We withheld onefold (test set) and estimated model parameters based on the remaining fourfold (training set). (2) We used model parameters from the model in step 1) to predict the withheld test set. (3) A 10-quantile binning was generated on the predicted values of the test set. Bins were ranked from low relative probability of selection (bin #1) to high relative probability of selection (bin #10). A model with good predictive performance tends to have successively more willow ptarmigan locations in higher ranked bins. (4) A statistical metric of model performance was assessed by spearman rank-correlation between bin-rank and the count of used locations in each bin. Strong positive correlation coefficient suggests good predictive performance [7]. We also performed the four cross-validation steps described above on four geographical regions (blocks, c.f. [46]) instead of fivefold. The regions were drawn up by similarities in climatic conditions, and willow ptarmigan population dynamics within regions are synchronized [22].

#### Predicting resource selection functions across Norway

We developed a predictive resource selection map based on the most parsimonious model. In order to do so we had to convert all raster maps to 30 × 30 m resolution using program GRASS with the function «r.resamp.stats» [36], through RStudio [30] with library «rgrass7» [37]. Then we estimated the resource selection function for each 30 × 30 m cell by putting selection coefficients ($\beta_{1}$, $\beta_{n} \ldots$) from the selected model and the raster values (i.e. explanatory variables $x_{1}$, $x_{n \ldots }$) into Eq. (1). This procedure creates a new raster containing RSF values for each cell. The predicted RSF values where scaled so that they were bounded between zero and one by dividing by the maximum RSF value. Following the recommendations in Morris et al. [47], we mapped the RSF values based on the same quantile bins as we used in the k-fold cross-validation procedure. Hence the map classification ranges from category 1 (low relative probability of selection) to 10 (high relative probability of selection). We calculated the percentage distribution of each RSF-bin across the whole of Norway, within transect buffers and among the used willow ptarmigan locations. After producing the RSF-map for the whole of Norway, we performed another validation procedure using an independent willow ptarmigan location dataset from the Global Biodiversity Information Facility (gbif) [48]. We downloaded 5787 observations of willow ptarmigan that were recorded by ornithologists in Norway during June to September from 2000 to 2017 [48]. We extracted the RSF-bin rank for each observation using GRASS [36] and the function «r.what» through RStudio [30] with library «rgrass7» [37] and counted the number of observations in each RSF-bin. Some observations (1258) were in empty map-cells (missing data for one or more of the predictor maps used in the modelling or were in non-habitat cells) so a total of 4529 independent observations could be linked to an RSF-bin. We calculated spearman rank correlation between the RSF-bin rank and area-adjusted number of observations in each bin. Area-adjusted number of observations was the number of observations in each bin divided by the availability of that RSF-bin in Norway (c.f., Table 6).

## Results

### Model selection and validation

In our model set, two models were equally supported by the data when considering the trade-off between model fit and number of parameters (i.e. ΔAIC < 2: Table 3). Both models included vegetation type (16 categories), deviation from the timberline (linear and quadratic terms) and slope (linear and quadratic terms). The highest ranked model included aspect as a categorical variable, whereas the second ranked model did not include any terms for aspect. Hence, the second ranked model had fewer parameters (21 vs. 25) and was considered as the most parsimonious model. K-fold cross-validation based on the most parsimonious model showed high and significant correlation for all fivefold (Table 4a), with a mean cross-correlation r = 0.96. This suggest that willow ptarmigan locations tend to be in high ranked habitats predicted by the model. All correlations from the regional cross-validation were significant, but the coefficients were much higher for the eastern, southern and western regions than for northern region (Table 4b).

**Table 3:** The 95% confidence set for models with ΔAIC < 5

Model	K	AIC	ΔAIC	AIC_wt_
Vegetation type + Timberline + Timberline^2^ + Slope + Slope^2^ + Aspect Categorical	25	53,190.3	0	0.42
Vegetation type + Timberline + Timberline^2^ + Slope + Slope^2^	21	53,190.65	0.34	0.35
Vegetation type + Timberline + Timberline^2^ + Slope + Slope^2^ + Aspect North–South	22	53,192.49	2.19	0.14
Vegetation type + Timberline + Timberline^2^ + Slope + Slope^2^ + Aspect North–South + Aspect East–West	23	53,193.26	2.95	0.09

Model probabilities sum to one

**Table 4:** Spearman rank correlations between RSF bin ranks and count of used locations in each RSF bin for (a) fivefold portioning of the data and (b) spatial blocking of the data into four geographical regions

(a)	Fold	Rho	p-value
	1	0.976	< 0.001
	2	0.988	< 0.001
	3	0.952	< 0.001
	4	0.939	< 0.001
	5	0.952	< 0.001

(b)	Region	Rho	p-value
	Centre	0.964	< 0.001
	North	0.636	0.048
	East	1.000	< 0.001
	West	0.988	< 0.001

### Selection coefficients

**Fig. 3:**
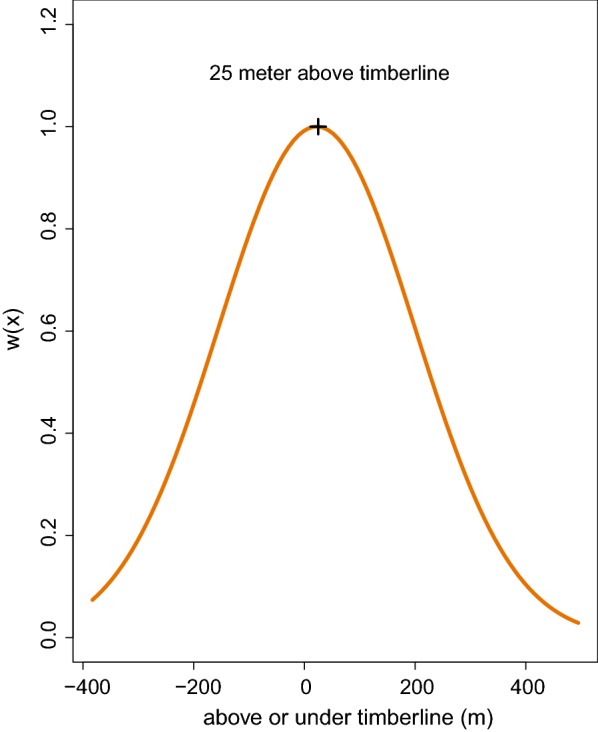
Relative probability of selection w(x) as a function of deviation from the timberline and deviation from the timberline2. The “plus” marker shows the polynomial inflection point

**Fig. 4:**
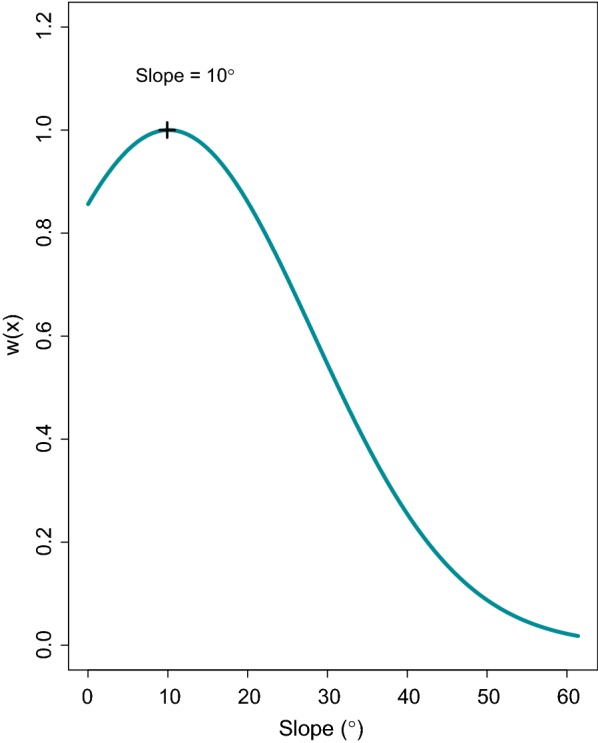
Relative probability of selection w(x) as a function of slope and slope2 in degrees. The “plus” marker shows the polynomial inflection point

Willow ptarmigan generally selected for areas around the timberline (Table 5). More specifically, selection increased from lower elevations towards the timberline and decrease above a polynomial inflection point at 25 m above the timberline (Fig. 3). Willow ptarmigan also select for intermediate slopes (Table 5) whit an inflection point at 10°, whereas steeper slopes were increasingly avoided (Fig. 4).

**Table 5:** Parameter estimates from the most parsimonious model

Variable	Category	Estimate	SE	p-value
Vegetation type	*Exposed alpine ridges (intercept)*	− *1.869*	*0.145*	< *0.001*
	*Bilberry*-*low fern birch forest*	*0.480*	*0.154*	*0.002*
	*Wet mires, sedge swamps and reed beds*	*0.493*	*0.170*	*0.004*
	*Fresh heather and dwarf*-*shrub communities*	*0.825*	*0.142*	< *0.001*
	*Graminoid apline ridge*	*0.460*	*0.161*	*0.004*
	*Herb*-*rich meadows*	*1.126*	*0.152*	< *0.001*
	*Tall*-*grown lawn vegetation*	*0.830*	*0.150*	< *0.001*
	*Crowberry birch forest*	*0.731*	*0.159*	< *0.001*
	*Lichen*-*rich birch forest*	*0.346*	*0.161*	*0.032*
	Lichen-rich heathland	− 0.233	0.168	0.166
	*Heather*- *and grass*-*rich early snow patch communities*	*0.492*	*0.150*	*0.001*
	*Heather*-*rich alpine ridge*	*0.599*	*0.143*	< *0.001*
	*Lowland forest*	*0.355*	*0.148*	*0.017*
	Bryophyte late snow patch vegetation	0.273	0.160	0.089
	Gras and dwarf willow snow-patch vegetation	0.026	0.180	0.883
	*Ombrotrophic bog and low*-*grown lawn vegetation*	*0.928*	*0.146*	< *0.001*
*Deviation from timberline*		*0.071*	*0.018*	< *0.001*
*Deviation from timberline* ^2^		− *0.257*	*0.018*	< *0.001*
*Slope*		*0.159*	*0.031*	< *0.001*
*Slope* ^2^		− *0.193*	*0.034*	< *0.001*

Variables in *italic* is significant with p-value < 0.05. Categorical variables in *italic* are significant relative to the reference class (exposed alpine ridges)

Among the 16 vegetation classes (Table 5), there was a clear selection for herb-rich meadows and clear avoidance for lichen-rich heathland relative to other habitat types. Four other vegetation types had parameter estimates above average; ombrotrophic bog, tall-grown lawn vegetation, fresh heather- and dwarf-shrub and crowberry birch forest. Other vegetation types with selection rates well below average included exposed ridges, dwarf willow snow patches, late snow patches, lichen-rich birch forest, and lowland forest classes.

### Predicting resource selection functions across Norway

We predicted relative selection probability for willow ptarmigan across Norway by calculating an exponential RSF (formula 1) using selection coefficients (Table 5) estimated based on the most parsimonious model in the candidate set (Fig. 1b). This model predicts that almost 60% of the mapped area (excluding non-habitat, see methods) is located in the lowest RSF-bin (i.e. low habitat suitability). Only 2% is located in the highest ranked RSF-bin (i.e. highest habitat suitability). If we take the lowest RSF-bin to be unsuitable for willow ptarmigan and the remaining bins 2–10 are increasingly suitable, about 41% of Norway is suitable for Willow ptarmigan (when not considering non-habitat). Further, for the area covered by the line transect surveys (i.e., areas within 200 ms from the transect lines), only 12% is predicted to be unsuitable and about 88% suitable for willow ptarmigan. For percentage distribution of RSF bins, see Table 6. Spearman rank-correlation between RSF-bin rank and number willow ptarmigan observations from gbif adjusted for availability of bin-ranks in Norway (c.f., Table 6 column 1) was high and significant (rho = 0.857, p = 0.002). This suggest that the model also have high predictability on independent data.

**Table 6:** Percentage cover of RSF-bins 1–10; across the whole of Norway, within the surveyed area (i.e., within 200-m buffers) and the distribution of willow ptarmigan locations across RSF bins, respectively

RSF-bin	Norway (%)	Surveyed area (%)	Willow ptarmigan locations (%)
1	59	12	5
2	12	12	7
3	7	11	8
4	5	10	10
5	4	10	10
6	3	10	11
7	3	9	11
8	3	9	12
9	2	9	13
10	2	9	14

Note that the values are rounded

## Discussion

The habitat suitability model developed here for willow ptarmigan was highly predictive according to both the k-fold cross-validation and validation against independent data, and therefore identify important habitats for willow ptarmigan during late summer/early autumn in Norway. Willow ptarmigan in general selected for herb-rich meadows and avoided most strongly lichen-rich heathland. Inspection of all vegetation type coefficients show that the general trend is selection for alpine vegetation types and bogs with abundant field layer and avoidance for sparsely vegetated alpine vegetation types and lowland forest vegetation types. In addition to selection and avoidance for different vegetation types, willow ptarmigan selected for areas around the timberline and for intermediate slopes.

Several other studies have described habitat selection in willow ptarmigan during different seasons [2, 10, 17–20, 49]. We describe habitat selection during late summer/early autumn. Kastdalen et al. [10] used similar data from August surveys, but for a much smaller area and using finer scaled vegetation data. In the study by Kastdalen et al. [10], willow ptarmigan selected rich bogs close to willow- and dwarf birch thickets and they avoided dry and poor open areas like heath. Our results coincide with Kastdalen et al. [10] with respect to both selection and avoidance. First, we found relatively strong selection for open alpine vegetation types with dense field layer and for bogs. Second, we found that willow ptarmigan avoided lichen-rich heath and other sparsely vegetated alpine areas such as ridges and late snow-patch vegetation. Similarly, studies of willow ptarmigan brood movements during summer in Northern Norway [20] and central Norway [19] also found that young broods used heaths less frequently than expected from the availability. In both studies, broods selected strongly for rich bogs. The vegetation types we used have a broad definition, so it is difficult to separate important small-scale habitat features. We can however, separate vegetation types that potentially contain important habitat features for willow ptarmigan (e.g. willow thickets, bogs and dwarf birch thickets [10, 17–20]). Willow thickets occur in bilberry-low fern birch forest and crowberry birch forest, on tall-grown sedge bogs and in fresh heather and dwarf-shrub communities. Among the bogs, both tall-grown sedge bogs and ombrotrophic bogs are high ranked vegetation types. Wet bogs and swamps is characterized by water level on the surface throughout the growing season and has an intermediate selection rank. Dwarf birch thickets occur in bilberry-low fern birch forest, fresh heather and dwarf-scrub, tall-grown sedge bogs, lichen-rich birch forest, heather- and grass-rich leeside. Dwarf birch do also occur on lichen-rich heathland and heather-rich alpine ridges, but in these sparsely vegetated and wind-exposed vegetation types, dwarf birch occurs more sparsely than in the latter types. The highest ranked vegetation type—herb-rich meadows—do not typically include willows or dwarf birch. It is however, characterized as the most nutrient rich vegetation type in the alpine region often with a stable water supply [34]. This might suggest that herb-rich meadows interact with other preferred vegetation types such as bogs or fresh heather and dwarf-shrub communities. Ehrich et al. [18], Henden et al. [50] advocate the importance of willow tickets for willow ptarmigan occupancy and both studies were carried out in low arctic tundra (e.g. same as the northern region in this study). Unfortunately, our vegetation maps cannot separate this vegetation structure, but it is possible that such strong selection for willow thickets in the arctic tundra is a special adaptation to a different environment.

Although the model is highly predictive according to the k-fold cross-validation on independent test data, the regional cross-validation for the northernmost region is barely significant. One reason for this low correlation could be the relatively low number of survey areas in this region. Only 32 survey areas out of a total of 179, and only 1096 used locations out of a total of 12,146 were in the northernmost region. However, this explanation might not be supported because the model was highly predictive in the western region where the observations are even fewer (14 survey areas and 426 observations). More likely, either willow ptarmigan selects differently in the low arctic tundra than in alpine tundra, or the broadly defined vegetation types in our vegetation map has different forms in low arctic tundra in north compared to areas further south. Another possible explanation can be that the relative probability of selection for a certain habitat type change with the availability of that habitat type (e.g., as a functional response [38, 51]). Hence, the availability of certain habitat types might vary from north to south. Since we use marginal selection coefficients applied to the whole of Norway, we are not able to account for such effects. Although the correlation is significant (Table 4b), building of a specific model for the northern region could result in a better predictive RSF for this region.

We followed the recommendation for mapping RSFs in Morris et al. [47] and partitioned our RSF predictions into 10 quantile bins before predicting the RSF across Norway. The bin ranks stretch from low relative probability of selection (1) to high relative probability of selection (10). The k-fold cross validation results suggest the model is highly predictive in describing the relative change in probability from bin 1 to bin 10. About 60% of Norway, 5% of the willow ptarmigan locations and 12% of the surveyed area (within buffers) are in lowest RSF bin (Table 6). Our data originate from a distance-sampling scheme, where the primary aim was to estimate willow ptarmigan densities in survey areas. Thus, data is systematically collected in areas where willow ptarmigan is expected to occur. When we predict across Norway, this will in turn lead to challenges in portion habitats with *no probability of selection* (e.g., forest and meadow habitats at low elevations, and coastal areas in southern latitudes and far from willow ptarmigan core habitats) since such areas were not surveyed. Based on this, we expect the lowest RSF bin to both include areas of *low relative probability* of selection that might be in or adjacent to alpine areas and *no probability* of selection (i.e. sites are far from willow ptarmigan core areas).

In general, animals are more abundant in habitats that are selected most strongly, and Boyce et al. [52] proposed that abundance can be estimated directly from habitat selection models for populations at the carrying capacity or for populations following an ideal free distribution. However, despite this and other studies documenting that willow ptarmigan select for specific habitat features [2, 10, 17–19, 49], Kvasnes et al. [53] found no clear relationship between willow ptarmigan density estimated pre-harvest (in August) and proportion of different habitat categories within survey areas. As also noted by Boyce et al. [52], Kvasnes et al. [53] suggested that other factors that are not directly related to habitat also influences the abundance. Willow ptarmigan population densities in the study of Kvasnes et al. [53] were generally lower than historic densities [54, 55]. In addition, different harvest strategies [56] and varying predation rates can have great influence on population densities in willow ptarmigan, both of which can vary independent of habitat composition. Habitat selection can also be affected by social interactions such as conspecific attraction [57], which is also suggested as a possible factor influencing the distribution of willow ptarmigan [58]. In our model, we assume that the explanatory variables have the same effect across all survey areas. Thus, we predict relative probability of selection for willow ptarmigan regardless of density and factors affecting density such as harvest rates, predator densities and human infrastructure.

Our RSF is based on data collected in August, so the resource selection map we developed is restricted to a short period of time. It is the phase when chicks are fledged, still accompanied by the adults, but only a few weeks prior to harvest. About 120,000–200,000 willow ptarmigans are shot each year in Norway (Statistics Norway, http://www.ssb.no/jord-skog-jakt-og-fiskeri), so an RSF based on data from August combined with density estimates from the same period can be a useful tool for harvest management. Further, the high and significant correlation for the independent validation-dataset recorded from June to September suggests that the RSF might be a useful tool to identify willow ptarmigan habitat potential during most of the snow-free season. An RSF based on winter locations of willow ptarmigan could also be useful in land-use management since most of the new infrastructure development in alpine and tundra areas is building of recreational facilities such as cabin villages. Cabin villages is mainly located in sub-alpine birch forests and boreal forests [59] and mountain birch is one of the main food items for willow ptarmigan during winter [60].

## Conclusions

We found that willow ptarmigan selects for vegetation types with dense field layer and bogs at intermediate slopes around the timberline. Selection for vegetation types coincide with previous small-scale habitat selection studies on willow ptarmigan. This is the first attempt to assess and quantify habitat selection for willow ptarmigan at a large scale using data from line transect distance sampling surveys, and model validation show that the model is highly predictive. Based on our RSF it is predicted that c. 60% of the land area in Norway is in the lowest RSF bin. This implies that these areas have very low probability of selection by willow ptarmigan compared to higher ranked RSF bins. Spatial variation in the model’s predictive capability suggest that habitat selection vary from north to south. Despite the conservation status *near threatened* (NT) in Norway, willow ptarmigan is a highly valued game species, and c. 50,000 register to hunt ptarmigan each year (Statistics Norway, http://www.ssb.no/jord-skog-jakt-og-fiskeri). There is also a continued demand for hydroelectric power installations, recreational facilities and roads within willow ptarmigan areas. The consequences are loss of habitat and thus a reduced potential carrying capacity for willow ptarmigan and other alpine species. The resource selection map we have developed predicts relative probability of selection during summer/early autumn and can be a useful management tool in the conservation of habitats across Norway. We recommend that the RSF is considered when planning harvest quotas and when planning habitat interventions in willow ptarmigan ranges. The latter can potentially also benefit other species with overlapping distributions.

## Abbreviations

RSF: resource selection function is a function that is proportional to the probability of selection by an animal
DEM: digital elevation model, a raster containing pixels with values representing elevation
RET: regional empirical timberline, a raster containing pixels with values representing the location of the timberline in meters above sea level
GBIF: Global Biodiversity Information Facility
